# Nutraceuticals for the Treatment of IBD: Current Progress and Future Directions

**DOI:** 10.3389/fnut.2022.794169

**Published:** 2022-06-06

**Authors:** Quan-Yao Ban, Mei Liu, Ning Ding, Ying Chen, Qiong Lin, Juan-Min Zha, Wei-Qi He

**Affiliations:** ^1^Department of Oncology, The First Affiliated Hospital of Soochow University, Jiangsu Key Laboratory of Neuropsychiatric Diseases and Cambridge-Suda (CAM-SU) Genomic Resource Center of Soochow Medical School, Suzhou, China; ^2^Department of Gastroenterology, The Affiliated Wuxi Children's Hospital of Nanjing Medical University, Wuxi, China; ^3^State Key Laboratory of Pharmaceutical Biotechnology, Nanjing University, Nanjing, China

**Keywords:** Inflammatory bowel disease (IBD), nutraceutical, immunity, intestinal mucosal barrier, short-chain fatty acids (SCFA), polyphenols, Vitamin D

## Abstract

Inflammatory bowel disease (IBD) is a chronic relapsing-remitting inflammatory disease of the gastrointestinal tract. Patients are usually diagnosed in adolescence and early adulthood and need lifelong treatment. In recent years, it has been found that diet plays an important role in the pathogenesis of IBD. Diet can change intestinal barrier function, affect the structure and function of intestinal flora, and promote immune disorder, thus promoting inflammation. Many patients believe that diet plays a role in the onset and treatment of the disease and changes their diet spontaneously. This review provides some insights into how nutraceuticals regulate intestinal immune homeostasis and improve intestinal barrier function. We reviewed the research results of dietary fiber, polyphenols, bioactive peptides, and other nutraceuticals in the prevention and treatment of IBD and sought better alternative or supplementary treatment methods for IBD patients.

## Introduction

Inflammatory bowel disease is a chronic gastrointestinal inflammatory disease that includes Crohn's disease (CD) and ulcerative colitis (UC). The pathogenesis of IBD is not clear, but it is thought to be caused by a complex interaction of genetic, environmental, immunologic, and microbial factors (1, 2). Most patients are diagnosed during puberty and early adulthood, and disease relapse and remission occur alternately. The main symptoms include abdominal pain, diarrhea, nausea, rectal bleeding, and weight loss, which have a serious impact on the quality of life of patients. Therapeutic drugs for IBD include 5-aminosalicylic acid (5-ASA), corticosteroids, immunosuppressants, and biological agents. However, the current treatment methods cannot completely cure the disease (3). Moreover, evidence has shown that conventional drugs hurt the growth and development of children with IBD (4). Therefore, it is necessary to deeply study the pathogenesis of IBD and explore better treatment methods.

In developed countries, the number of patients with IBD is increasing exponentially, which places an increasing economic burden on the health care system (5). Interestingly, this disease, which only occurred in developed countries in the past, has increased rapidly in recent years in developing countries that have experienced changes in westernized lifestyles (6, 7). A research report showed that a Western diet characterized by red meat, sugary desserts, high-fat foods, and refined grains may increase the risk of intestinal inflammation. In contrast, a Mediterranean diet (MD) rich in fruits, vegetables, whole grains, poultry, and fish may reduce the incidence of IBD (8). This review discussed the role of diet in the onset of IBD. We also reviewed the potential role of nutritional supplements in preventing and treating IBD.

## The Role of Diet in the Onset of IBD

Although the pathophysiological mechanism of IBD is not completely clear, a large number of studies have shown that diet plays a key role in the occurrence of IBD by regulating intestinal barrier function, intestinal immune function and intestinal microflora. In addition, increasing evidence indicates that diet plays an important role in the development of IBD among genetically susceptible individuals.

### Effects of Diet on Intestinal Barrier Function

**Figure 1:**
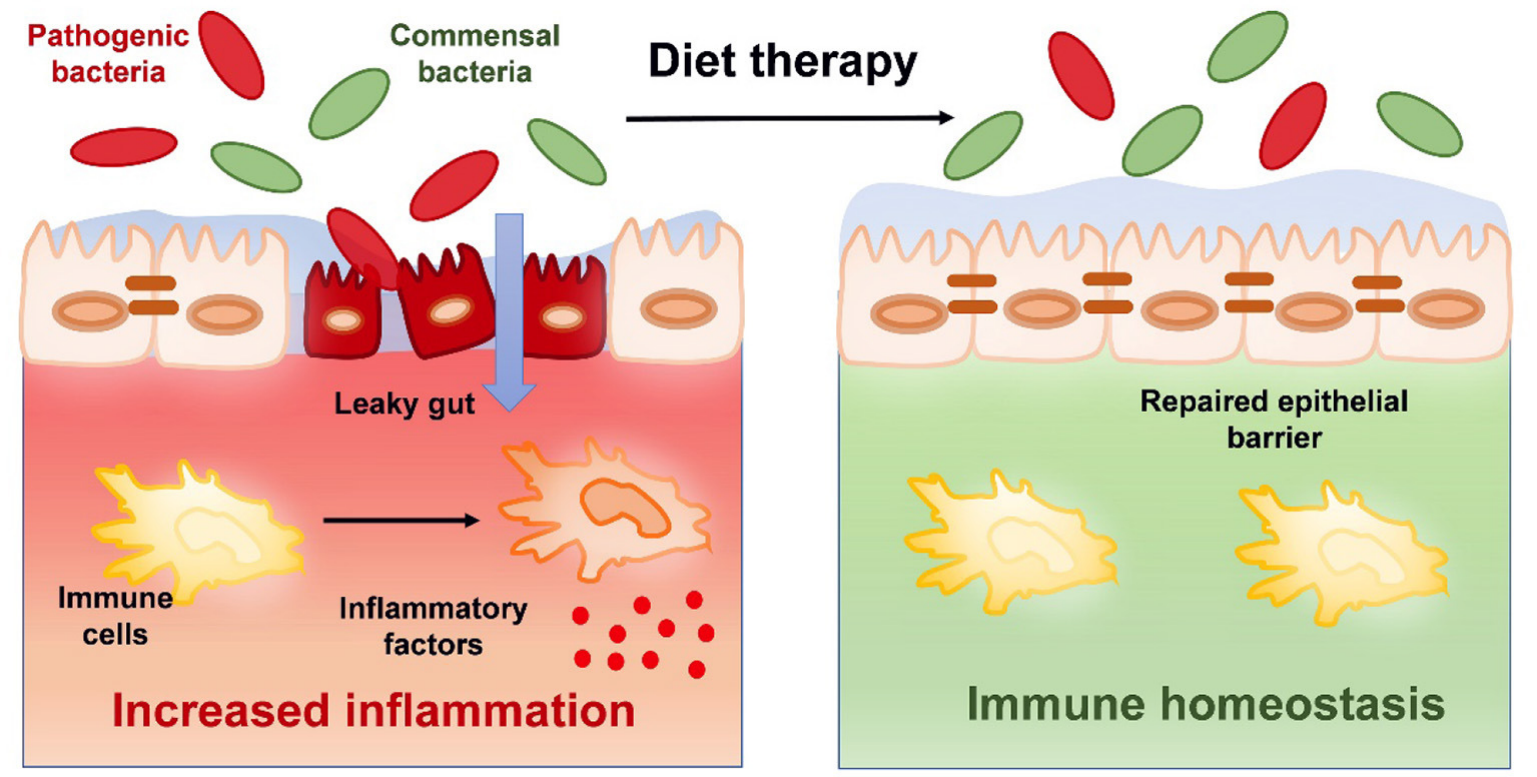
The therapeutic effects of diet on intestinal barrier function and immune homeostasis in IBD.

The intestinal tract plays a physiological role by absorbing water and nutrients and needs to resist the invasion of harmful substances in the intestinal cavity (9). As the digestive part of the human body, the intestinal cavity is exposed to antigenic components in food and a variety of microorganisms. As a physical barrier containing biochemical and immune components, the intestinal epithelium separates the host from the external environment. The physical components of the mucosal barrier include epithelial cells closely connected by tight junctions and mucus secreted by epithelial cells (10). The integrity of the intestinal barrier is very important to stabilize the intestinal environment and prevent microbial invasion. Tight junctions play an important role in maintaining the integrity of the intestinal epithelium, and their expression is regulated by various signaling pathways (11) (Figure 1). We found that under pathological conditions, proinflammatory cytokines activated myosin light chain kinase (MLCK) and triggered the contraction of the perijunctional actomyosin ring (PAMR), which led to an increase in tight junction permeability (12, 13).

IBD is characterized by the high penetration of bacteria into the mucus layer and the increase in barrier permeability. The damaged intestinal mucosal barrier leads to pathogen invasion and the local abnormal activation of immune cells, and inflammation further aggravates intestinal mucosal damage (14). Genetic studies support IBD susceptibility genes encoding proteins, which play a key role in intestinal barrier function, and emphasize the involvement of the intestinal barrier in the pathogenesis of IBD (15).

Dietary fiber in vegetables and fruits is an important substrate for producing short-chain fatty acids such as butyric acid. Butyric acid plays an important role in reducing inflammatory reactions by inhibiting the transcription of cytokines and increasing the differentiation and number of lamina propria cells (16). In animal studies, butyric acid can also decrease intestinal permeability and enhance the expression of tight junction proteins by upregulating defensins and antibacterial peptides (17, 18). In contrast, low dietary fiber may lead to increased catabolism and permeability of the mucosal layer and increase the contact between bacteria and epithelial cells (19).

A high-fat (HF), high-sugar (HS), and low-fiber diet may have synergistic effects. The HF/HS diet has been proven to decrease the expression of *Muc2*, increase intestinal permeability, and induce the production of mucus in goblet cells. Similarly, these diets reduced the production of butyric acid and the expression of the butyric acid GPR43 receptor, which was absent in CD (20).

### Effects of Diet on Mucosal Immune Function

The intestinal barrier, microbial community, and immune system interact with each other in a highly integrated and interdependent way to maintain intestinal homeostasis. A Western diet is characterized by a high-fat diet, which often leads to obesity and has multiple effects. A Western diet can cause an imbalance of Th17 cells and Treg cells in the body, reduce IgA antibody secretion by reducing B cell activation, and finally reduce intestinal defense. A high-fat diet has been proven to increase the expression of TNF-α and IFN-γ and decrease the levels of Tregs in the colon (Figure 1) (21). Moreover, obesity caused by a high-fat diet increases the secretion of inflammatory mediators, and some adipocytokines, such as leptin, in adipose tissue activate the intestinal immune response and promote the occurrence of UC. In addition, free fatty acids reduce the number of lymphocytes and impair the immune function of the intestinal tract. A long-term high-fat diet reduced short-chain fatty acids and tretinoin, and dendritic cells triggered CD4^+^ T-cells to differentiate into Treg cells, thus destroying the intestinal immune barrier (22). A high-fat diet can lead to insulin resistance, affect the balance of fatty acids, reduce the number of lymphocytes, leading to inflammation of the intestinal epithelium, change intestinal permeability, and contribute to the production of sulfur, which in turn changes intestinal microorganisms by promoting the growth of sulfate-reducing bacteria (23). It should not be ignored that different fat sources play different roles in promoting inflammation. Devkota et al. reported an increase in colitis severity after exposure to a milk fat diet but not to an isocaloric polyunsaturated fatty acid diet or a low-fat diet in IL-10 knockout mice (23).

The α-amylase and trypsin inhibitors found in wheat may have proinflammatory effects. They have the highest concentrations in gluten-containing cereals, including bread, pasta, ice cream and other western foods. They are activators of human dendritic cells and macrophages, which can cause inflammation of the rodent intestinal tract (24, 25). Western diets are usually rich in fat and sodium. A high-salt diet can also affect mucosal immune function. Dietary sodium intake promotes the development of IBD through IL-23 and IL-23 receptor-mediated activation of pathogenic proinflammatory Th17 cells. In addition, sodium can inhibit immune tolerance by inhibiting the immunosuppressive function of FOXP3 and Treg cells, thus promoting the occurrence of inflammation. Sodium may also lead to the activation of proinflammatory macrophages (26).

### Effects of Diet on Intestinal Microbes

The intestinal microflora is a large collection belonging to four phyla: Firmicutes, Bacteroidetes, Proteobacteria, and Actinobacteria. It is generally agreed that IBD is related to the composition and metabolic changes in intestinal microflora (dysbiosis). Compared with healthy subjects, IBD patients showed a decrease in intestinal microbial diversity, accompanied by a decrease in anti-inflammatory flora (2). The intestinal flora is regulated by many factors, such as genes, age, stress, diet, and antibiotics. Changes in long-term diet patterns and short-term nutrition intake can lead to changes in intestinal flora (27). In animal models, mice fed a high-fat, high-sugar, low-fiber diet had an increase in the Phylum Firmicutes and a decrease in the Phylum Bacteroidetes (28). High sodium content can regulate the composition and function of intestinal microbes, that is, reduce the relative abundance of Lactobacillus and the level of butyrate short-chain fatty acids and promote the proinflammatory state in the intestine (29). The increase in heme iron intake in the diet is also related to the significant changes in intestinal flora composition, in which the ratio of Gram-negative bacteria to Gram-positive bacteria will increase (30). Data from global cross-sectional studies indicate that dietary fiber can increase the diversity of intestinal flora (27). A Dutch cohort study demonstrated that carbohydrate intake, including high calorie intake and consumption of sugar-sweetened beverages, was negatively correlated with intestinal microbial diversity (31). In contrast, characteristics of the Mediterranean diet, such as the intake of fruits, vegetables, and red wine, were associated with increased diversity of gut microbes. Meanwhile, the intake of red wine is related to an increase in Bacillus, which is believed to have anti-inflammatory properties in IBD patients (32). Recent studies have shown that the intestinal flora regulates host immunity through metabolites, that is, small molecules produced by microorganism metabolism. Apart from directly regulating immunity, diet can also indirectly regulate host immunity through metabolites produced by fermentation of dietary substrates (4). Taking probiotics is one of the strategies to prevent and treat IBD because probiotics can regulate the intestinal microflora, the mucosal immune system, and the production of metabolites (33). A recent study showed that oral administration of a new probiotic strain, *L. lactis* ML2018, has therapeutic potential for treating IBDs (34).

### Interactions Between Diet and Genetic Susceptibility in IBD

A European cohort study confirmed the interaction between iron and heme iron intake and rs1801274, a coding body in the susceptibility locus FCGR2A of UC (35). The increase in heme iron intake was related to a decrease in UC risk in women with the GG genotype, while the increase in heme iron intake was related to a triple increase in UC risk in women with the TT genotype (36). There was also an interaction between potassium intake and IBD-related IL-21 variant RS7657746. Increased dietary potassium intake was inversely associated with UC risk in participants with the IL-21 GG genotype but not in participants with the AG or GG genotype (37–39). Finally, data from the European cohort also showed that mutations in CYP4F3, a cytopigmented P450 enzyme encoding PUFA metabolism, may alter the relationship between the intake of n-3 and n-6 short-chain fatty acids and the risk of UC. A high n-3:n-6 intake diet was associated with a lower risk of UC in women with the GG or AG genotype but not with the AA genotype (40).

## Diet as a Therapeutic Intervention

Recently, there has been a resurgence of interest in the potential of dietary intervention for the treatment of IBD. A cross-sectional study has shown that IBD patients will change their dietary intake spontaneously and consciously consume vegetables, fruits, and whole grains in the standard recommended amount. Most of them believe that changes in dietary patterns can improve the symptoms of diseases, even without the guidance of a doctor (41). Accumulating evidence supports that dietary intervention for IBD patients can reverse immune and biological disorders and play a role in IBD treatment (6).

Several pediatric studies have demonstrated that exclusive enteral nutrition (EEN) can induce remission in 60 to 86% of Crohn's children, with significant reductions in inflammatory markers such as erythrocyte deposition (ESR) and C-reactive protein (CRP) (42–46). Clinical studies have shown that EEN given over some time can improve clinical symptoms and intestinal inflammation in CD, which is superior to corticosteroids in promoting mucosal healing in children with CD. EEN can also be used as an adjuvant therapy. In a retrospective study in Japan, EEN combined with Infliximab had a higher remission rate in patients with CD than EEN alone (47). In addition, EEN can even reduce the necessary surgery, improve surgical outcomes and reduce complications in the perioperative period. In a retrospective case–control study, 25% of patients planning to have selective surgery could avoid surgery or reduce postoperative complications such as anastomotic leakage and postoperative abscess, even if only 6 weeks of EEN were applied (48). In another study, compared with patients without EEN treatment, after 4 weeks of EEN and antibiotic treatment, patients with CD complications with an abdominal abscess had a lower probability of requiring surgery and a lower postoperative recurrence rate (49, 50). EEN has different types of enteral formulas, and the formula difference lies in the type and composition of fat and protein. A meta-analysis of 10 trials did not show any statistically significant difference between patients treated with an elemental diet and a semielemental diet or polymeric diet (51, 52). For patients with CD, there is no evidence that the special formula is superior to the standard polymeric formula (53). Increasing evidence shows that EEN has a direct anti-inflammatory effect and changes the intestinal microflora. However, the exact mechanism behind these changes is still unclear, and it is impossible to point out that some elements play an anti-inflammatory role. At present, the proposed hypotheses include avoiding the intake of harmful components, reducing the allergic load, and not containing nucleotides and anti-inflammatory lipids (51, 54).

Unfortunately, EEN has a limited effect on adult patients, as patients have to deal with monotonous food and taste fatigue. Furthermore, up to 50% of patients may require nasogastric tubes, and many patients might refuse to start therapy due to these issues. This has led to an interest in studying partial enteral nutrition (PEN) in combination with diet. Studies have shown that patients who use PEN combined with diet have a longer remission time than those who eat a normal diet. Similarly, Verma et al. found that PEN combined with diet can significantly reduce the relapse rate of IBD (55, 56).

In addition, dietary treatments for IBD include a Mediterranean diet, a low FODMAP diet, Crohn's Disease Exclusion Diet (CDED), and a special carbohydrate diet (SCD). The Mediterranean diet is mainly based on natural nutrients, including olive oil, vegetables, fruits, fish, seafood, and beans, plus a proper amount of red wine and garlic. In a recent study, the disease activity index and inflammatory markers were improved in the short term after patients with CD and UC consumed a Mediterranean diet (57). However, due to the harmful effects of alcohol on the gut, more evidence is needed to intervene red wine, an integral part of the Mediterranean diet (58, 59).

The low FODMAP diet refers to a diet with low fermentable oligosaccharides, disaccharides, monosaccharides, and polyols (FODMAPs). A low FODMAP diet can reduce irritable bowel syndrome (IBS)-like symptoms in patients with quiescent IBD (60). A randomized trial showed that a short-term low FODMAP diet improved the disease activity, fecal inflammatory markers and disease-specific quality of life of IBD patients (61). In another randomized controlled trial, compared to the control group, the severity score of irritable bowel syndrome in IBD patients with a low FODMAP diet decreased more, but there was no significant difference in inflammatory markers (62). Because the trial lasted only 4 weeks, the effect of a low FODMAP diet on IBD inflammation levels is uncertain.

CDED is designed to eliminate food ingredients such as certain animal fats and food additives, which can damage the intestinal mucosa. A prospective study demonstrated that compared with EEN alone, the combination of CDED and PEN showed similar efficacy but with better tolerance and longer remission duration (63). CEDE can rapidly reduce the inflammatory level of patients with IBD. In the third week of using the CEDE diet, the CRP level of nearly half of the patients returned to normal levels. In the sixth week, 76% of the patients achieved the normalization of clinical remission and inflammation index (64).

SCD is an exclusive diet consisting mainly of simple sugars, solid proteins, and fats. In a survey of patients on SCD, 33% were in remission after 2 months, and 42% were in remission after 6 months. SCD could reduce patient symptoms and inflammatory burden, and more exclusionary diets were related to a better resolution of inflammation (65).

Although SCD has considerable promise in the treatment of IBD, it is found that the intake of vitamin D and calcium is lower than the recommended daily intake. Therefore, we should pay attention to the risk of malnutrition. More studies are needed to explore the pros and cons of SCD in patients (66).

## Nutraceuticals for the Treatment of IBD

Many nutritional supplements derived from food have therapeutic effects and can improve host immunity (67). The principles of dietary interventions mentioned above are similar: it is worth considering increasing the intake of nutraceuticals that can induce and maintain remission in patients with IBD while limiting the intake of pathogenic food ingredients, which answers patients' most common questions: What should I eat? What should I avoid?

### Polyphenols

Polyphenols are a kind of phytochemical that are characterized by many phenolic groups. It exists in some common plant foods, such as cocoa, tea, soybeans, vegetables, and fruits (68). Polyphenols have received increasing attention from nutrition experts because of their important role in human health. It is well known that polyphenols can act as antioxidants to resist oxidative stress and alleviate neurodegenerative diseases and some cardiovascular diseases (68). Polyphenols also have anti-inflammatory, antitumor, and immunoregulatory properties (69, 70). In the past 10 years, the therapeutic effects of polyphenols in IBD have been increasingly studied. Researchers have found that polyphenols can reduce local oxidative stress, regulate the NF-κB and mitogen-activated protein kinase (MAPK) signaling pathways, and thus downregulate the secretion of inflammatory factors (71, 72). In a recent review, the therapeutic effect of resveratrol on IBD was attributed to reducing oxidative stress, inhibiting the activation of NF-κB, and reducing proinflammatory cytokines and prostaglandins (73). In addition, polyphenols can regulate the structure and function of intestinal flora, improve the stability of tight junctions and strengthen intestinal barrier function (74).

In a model of colitis induced by dextran sodium sulfate (DSS), supplementation with pomegranate extract increased the number of lactic acid bacteria and bifidobacteria and significantly decreased the number of *Escherichia coli* (*E. coli*) (75). Patients who took pomegranate extract showed an increase in actinomycetes. (75). Experiments have shown that grape seed extract can regulate the expression of essential proteins in the colonic tight junction complex, thus significantly reducing intestinal permeability (76). In another study, polyphenol extracts from red wine were shown to enhance the intestinal barrier by increasing the gene expression of essential tight junction proteins and inhibiting the formation of channels induced by inflammatory factors (77). Polyphenols and intestinal flora have a synergistic anti-inflammatory effect. Human studies have shown that polyphenols can increase the abundance of Lactobacillus and Bifidobacterium in the intestinal tract, and a large dose of polyphenols can reduce pathogenic microorganisms such as Parvimonas (72). A randomized controlled trial showed that red wine polyphenols could significantly regulate the intestinal flora structure, increase the number of healthy Bifidobacteria and reduce the level of inflammatory markers in serum (67). However, the intake of alcohol changes the composition of intestinal microflora, which will lead to intestinal dysbiosis (58). It was also found that alcohol intake was related to the decrease of abundance of main symbiotic microbial communities, the increase of intestinal permeability and disruption of intestinal immune response (59, 78). Red wine is low in polyphenols and high in alcohol. It might be very important to reduce alcohol consumption for the prevention and treatment of IBD. Therefore, more evidence, mainly from humans, is needed to support the use of red wine as an intervention in IBD.

#### Metabolites and Derivatives of Polyphenols

Most polyphenols cannot be directly utilized by the human body but are fermented by bacteria in the colon into metabolites with high anti-inflammatory characteristics and bioavailability. The measured concentration is several times higher than that of its precursor. Several studies have shown that polyphenol metabolites have a therapeutic effect on IBD. For example, the metabolite of caffeic acid, hydrocaffeic acid, can downregulate the expression of the proinflammatory factors TNF-α and IL-8 to reduce colitis (79). Studies have shown that the metabolites hydrolyzable tannins (HTs), especially urolithins, show strong structure-dependent anti-inflammatory properties (75). Isoflavone extracted from soybean is an important polyphenol substance, but its low water solubility restricts its practical application. Researchers have proven that soybean-derived isoflavone glycosides (SIFs), derivatives of isoflavones, show high bioavailability. *In vivo*, SIF improved the symptoms of colitis and tissue inflammation induced by DSS. *In vitro* experiments showed that pretreatment of LPS-activated RAW 264.7 macrophages with SIF alleviated inflammation (80). In the future, polyphenol metabolites and derivatives should be studied more because of their higher availability and biological efficacy.

#### Prevention of IBD Complications

Colitis-associated cancer (CAC) is a complication of IBD and is also a common cause of cancer-related death (81). At present, the preventive effect of drug therapy on CAC is poor. The side effects are obvious and are not suitable for long-term use. Berry, as a natural substance with low toxicity and side effects, has been used as a long-term preventive intervention in preclinical and clinical pilot studies (82–85). Accumulating evidence suggests that a variety of nutrients and biochemical substances found in strawberries and black raspberries, particularly anthocyanins, can control genome instability, reduce oxidative stress, and inhibit the production of inflammatory factors and NF-κB-related signaling pathways (86). Strawberry and black raspberry are promising methods for CAC chemoprevention by acting on targets related to inflammation-induced carcinogenesis. Studies have shown that resveratrol, a common polyphenol in berries, seems to inhibit the occurrence of tumors by regulating signal transduction mechanisms and programmed cell death (73). It has been reported that some Chinese herbal medicines containing polyphenols and other active ingredients regulate apoptosis by regulating various signal transduction mechanisms, thus playing a role in preventing CAC (87).

Iron deficiency is common in patients with IBD. Inflammatory factors can increase the expression of ferricin in the intestinal tract, thereby reducing the absorption of iron. Preliminary studies show that the polyphenol 7-hydroxy matairesinol (7-HMR) produced by plants can significantly reduce the gene expression of the low-iron regulator, thus improving the symptoms of iron deficiency (88).

#### Improvement of Bioavailability

For IBD, most of the side effects of drugs are caused by systemic administration. Drug delivery to specific inflammatory areas can reduce side effects and improve curative effects. Because of the low absorption of polyphenols, an increasing number of researchers have begun to explore the combination of polyphenols and nanocarriers. Patients with IBD are deficient in physiological lipids, and lipid-based oral preparations have been used in many diseases. Based on the anti-inflammatory properties of curcumin and the immunomodulation of lipids, the researchers designed three lipid nanocarriers loaded with curcumin and compared their therapeutic effects. The results showed that nanocarriers could reduce neutrophil infiltration and TNF-α secretion and reduce colitis induced by direct stimulation (89). Rosmarinic acid (RA) is an important polyphenolic substance. PEGylated RA-derived nanoparticles (RANPs) are a kind of RA-based nanodrug that have high stability and greatly improve the bioavailability of RA. Compared with the parent RA, intravenous injection of RANPs targeting the colon can significantly reduce DSS-induced colitis in a dose-dependent manner, showing broad application prospects. In addition, RANPs loaded with the corticosteroid dexamethasone showed better curative efficacy than RANPs alone, which indicated that traditional drugs and polyphenols may be used together to enhance the curative effect, and the new technology avoids the side effects of traditional drugs to some extent (90).

In summary, the combination of polyphenol metabolites and derivatives, as well as nanocarriers, significantly enhanced the therapeutic effect of polyphenols. Most studies on the mechanism of polyphenols remain at the experimental level, and more large-scale and clear human trials should be conducted to explore the therapeutic effects of polyphenols and their metabolites on IBD patients.

#### Polyphenols and Probiotics

Recently, experts suggested fermenting polyphenol-rich blackcurrant (BC) with lactic acid bacteria and adding it to yogurt to form a new type of healthy snack. Proteins in yogurt protect polyphenols by forming hydrophobic and hydrogen bond complexes. Dairy products also help improve the astringent taste of blackcurrant. In addition, adding polyphenols before fermentation promoted the growth of *Streptococcus thermophilus* and *Lactobacillus bulgaricus* in yogurt (91). In theory, fermentation can also improve the antioxidant capacity of blackcurrant (92). Polyphenols and probiotics act synergistically with each other, so BC yogurt has dual health benefits (Table 1). Although more research on human intervention is needed, it is believed that this new snack is the right choice to treat IBD (92).

**Table 1:** Effect of polyphenols on inflammatory bowel disease.

**Compound**	**Base material**	**Mode of action**	**Ref**
Polyphenols	Resveratrol	Reduced oxidative stress, inhibited NF-κB activation; Reduced pro-inflammatory cytokines and rostaglandins	(73)
	Pomegranate	Significantly reduced the number of *E. coli*	(75)
	Grapeseed	Significantly reduced intestinal permeability	(76)
	Red wine	Enhanced the intestinal barrier	(77)
Polyphenol metabolites	Hydrocaffeic acid	Downregulated expression of TNF-α and IL-8	(79)
	Urolithins	Showed structure-dependent anti-inflammatory properties	(75)
	Isoflavone glycosides	Reduced inflammation	(80)
Nanodrug based on polyphenol	Curcumin-loaded lipid-based nanocarrier	Reduced neutrophil infiltration, decrease the secretion of TNF-α	(89)
	RANPs	Reduced inflammation in a dose-dependent manner	(90)
Polyphenols and probiotics	Blackcurrant yogurt	Promoted the growth of *Streptococcus thermophilus* and Lactobacillus Bulgaricus showed the antioxidant capacity	(92)

*NF-κ,: nuclear factor-κB; TNF-α, TNF tumor necrosis factor–α; IL-8, interleukin-8; RANPs, rosmarinic acid-derived nanoparticles*.

### Dietary Fiber, Also Known as Prebiotics

Low fiber intake is an important characteristic of Western diets and is considered to be related to the risk of IBD. In epidemiological studies, compared to Western diets with high fat, high sugar, high starch and low fiber intake, a diet with high fiber intake tends to reduce the risk of IBD (93). In a cross-sectional study, the daily dietary fiber intake of children with IBD was lower than that of healthy subjects, far lower than the recommended intake (4). In a meta-analysis of prospective cohort studies, researchers found that dietary fiber intake was negatively correlated with CD risk (94). The researchers analyzed the dietary information of IBD patients collected in a large-scale study, grouped them according to different IBD subtypes, and minimized all kinds of information deviation. The results showed that compared with patients who avoided fiber intake, patients who did not avoid fiber intake had a lower risk of recurrence in the remission stage (95). Increasing evidence shows that increasing the intake of dietary fiber is beneficial to the management of IBD. Dietary fiber is a carbohydrate polymer that cannot be digested and absorbed by the intestinal tract. It is commonly found in fruits, vegetables, beans, and grains. In addition to natural fiber found in food, carbohydrate polymers derived from biochemical or synthetic techniques are also defined as fiber. Dietary fiber has attracted increasing attention because of its health benefits, and fiber supplements have also appeared on the shelves of goods. According to its water solubility, DF is divided into soluble dietary fiber (SDF) and insoluble dietary fiber (IDF). The former is more easily fermented by the colon. Diets containing a high proportion of soluble fiber, such as oats, barley, and fruits, are recommended to patients with IBD because they play a better role in prebiotics (6).

In addition, fibers with different solubilities ferment at different locations in the colon, and the amount of metabolites was proportional to the concentration of the fermenting bacteria. In animal models, mice fed different fiber types (short-chain oligofructose and cellulose) showed significant differences in the microbial community (27). Generally, foods such as fruits and grains are rich in many fiber types, contributing to the diversity of gut flora. The role of dietary fiber in the pathogenesis of IBD includes regulating the function of epithelial cells, changing the composition of the intestinal flora, and enhancing the function of the intestinal barrier. Dietary fiber has been proven to downregulate the expression of NF-κB in colon tissue, thereby inhibiting the activation of proinflammatory tumor necrosis factor-α (TNF-α) (96) (Table 2). Intestinal microorganisms produce harmful substances when fermenting amino acids or proteins, which may participate in the pathophysiological process of inflammation and colorectal cancer. Dietary fiber seems to inhibit this process, emphasizing the benefits of dietary fiber (97).

**Table 2:** Effect of dietary fiber on anti-inflammatory bowel disease.

**Compound**	**Base material**	**Mode of action**	**Ref**
IDF	Fruits	Altered the composition of the intestinal flora; Strengthened the intestinal barrier function; Downregulated NF-κB pathway	(96)
SCFA	Dietary fiber	Reduced intestinal permeability; blocked NF-κB pathway; Activated PPAR γ; Inhibited the growth of E. coli	(98–100, 102)
Neutral sugar side chains	Pectin	Stimulated the growth of Bacteroides; Contributed to the production of more propionic acid; Reduced the level of IL-1β and IL-6	(103)

*IDF, insoluble dietary fiber; NF-κB, nuclear factor-κB; SCFA, short-chain fatty acid PPARγ, peroxisome proliferator-activated receptor γ; E. coli, Escherichia coli; IL-1β, interleukin-1β; IL-6, interleukin-6*.

#### SCFA Regulation of the Immune Response

Short-chain fatty acids (SCFAs), metabolites of DF fermentation in the colon, have been shown to have immunomodulatory effects and regulate the intestinal flora (4). The decrease in SCFA-producing microflora is the main feature of intestinal microflora changes in IBD patients, and these metabolites are exhausted in the intestinal tract (2). SCFAs include acetate, propionate, and butyrate, in which butyrate is the main energy source of intestinal cells. Propionate can be converted into glucose through biochemical reactions and used as fuel (27). In addition, butyrate is believed to increase the expression of mucin and decrease intestinal permeability (98). The actin-binding protein synaptophysin (SYNPO) is an intestinal epithelial tight junction protein that has a positive effect on the maintenance of the epithelial barrier and wound healing. In the DSS-induced colitis model, SYNPO-deficient mice had a higher disease susceptibility. Butyrate induces the production of SYNPO by inhibiting histone deacetylases, thereby maintaining intestinal homeostasis (99). In summary, SCFAs are helpful to maintain the function of the colon epithelium and intestinal barrier.

As ligands of receptors on the surface of immune cells, SCFAs bind to G protein-coupled receptors (GPR41, GPR43, and GPR109A), blocking the NF-κB pathway and increasing the production of the anti-inflammatory factor IL-18. SCFAs also play an anti-inflammatory role by activating peroxisome proliferator-activated receptor γ (PPARγ) (98, 100). By inhibiting the activity of histone deacetylase, butyrate promotes the anti-inflammatory action of intestinal epithelial cells, macrophages, and dendritic cells (101).

A recent study showed that SCFA inhibited the growth of *E. coli*, a pathogenic commensal bacterium, and reduced its virulence under colonic conditions (102). Since *E. coli*, especially adhesive and invasive *E. coli*, is related to the pathogenesis of IBD disease, the treatment of *E. coli* is worth considering. Establishing a regional chemical microenvironment with a proper pH value and SCFA concentration is very important for the regulation of *E. coli*. There is evidence that SCFA has low oral compliance due to its unpleasant taste, and a more bioavailable drug delivery form is still to be developed in the future (98). It is suggested to ingest the substance producing SCFA, namely, dietary fiber, to achieve the expected therapeutic effects (Table 2). Probiotics and their metabolites, SCFAs, directly or indirectly regulate the intestinal microflora and immune system to participate in the steady state of the intestinal environment. Probiotics have been proven to have therapeutic effects on experimental colitis models in mice and clinical trials in patients with IBD (33).

#### SDF and IDF

In a large-scale long-term prospective study, researchers collected information from 170,776 women who participated in nurses' health studies and used a semiquantitative food frequency questionnaire to determine dietary information. They found that long-term intake of fiber from specific sources (mainly fruits) can reduce the risk of CD, while intake of fiber from grains, whole grains, and beans cannot (104). We speculate that this difference may be due to the additional effect of polyphenols in fruits (another nutritional product with therapeutic effects on IBD). Another potential mechanism is that fruits contain a high proportion of soluble dietary fiber. A recent study compared the therapeutic effect of soluble dietary fiber with that of insoluble dietary fiber. The results showed that they all significantly relieved the inflammatory symptoms of the mouse colitis model, including diarrhea, fecal hemorrhage, and weight loss. SDF specifically increased the abundance of lactic acid bacteria and contributed to the promotion of the diversity of intestinal flora (96). Inulin, a well-known SDF, enhances the growth of indigenous lactobacilli and/or bifidobacteria, conferring positive effects on gut health. In rat IBD models and human subjects, inulin reduced intestinal mucosal damage and relieved intestinal inflammation (52). Pectin is rich in soluble dietary fiber in fruits. Studies have shown that pectin with more side chains can stimulate the growth of Bacteroides and help produce more propionic acid than pectin with fewer side chains. In addition, the neutral sugar side chain of pectin significantly reduced the levels of IL-1β and IL-6 in a microorganism-independent manner, thereby reducing colonic inflammation (103) (Table 2).

### Vitamin D

Vitamin D is a fat-soluble steroid hormone that is divided into vitamin D2 and vitamin D3. The main source of the human body is vitamin D3 synthesized by keratinocytes in the sun. When lack of sunlight leads to insufficient endogenous vitamin D production, vitamin D can be directly taken in through a daily diet or nutritional supplement. Only activated vitamin D plays a physiological role. Vitamin D is absorbed in the small intestine, transported to the liver and converted into 25(OH)D, finally forming its active form in the kidney in 1,25 dihydroxyl vitamin D(1,25 (OH)2D). Vitamin D, as a pleiotropic hormone, not only contributes to the absorption of calcium and phosphate but also plays a vital role in maintaining intestinal immune homeostasis and intestinal barrier function (105).

#### Vitamin D/VDR Axis

Vitamin D and vitamin D receptor (VDR) combine and transcribe related genes, which play a role in immune regulation. When microorganisms invade epithelial cells, vitamin D/VDR signal transduction activates various immune cells, which ensures the elimination of bacteria. Then, vitamin D regulates the expression of proinflammatory factors by promoting Treg cell production of IL-10 and inhibiting Th1/Th17, thus regulating the excessive immune response (106–108). In this way, vitamin D can limit the inflammatory reaction and avoid mucosal injury. Therefore, the vitamin D/VDR axis is considered to be helpful to maintain intestinal immune homeostasis. In addition, the vitamin D signaling pathway regulates the tight junctions of intestinal epithelial cells, promotes the expression of antibacterial peptides, and improves intestinal barrier function (109).

#### Vitamin D and IBD Prognosis

Vitamin D deficiency is a predictor of poor prognosis for IBD. Data show that low vitamin D levels in IBD patients are related to an increase in disease activity, hospitalization, and medication (110, 111). In a recent meta-analysis and systematic review, the author thinks that increasing vitamin D levels could reduce the recurrence of IBD disease and improve the quality of life of patients, such as improving bone pain and self-health. This conclusion is consistent with previous studies (105). A previous randomized controlled trial showed that low vitamin D levels before diagnosis were not associated with IBD, while postdiagnosis low vitamin D levels were associated with IBD. The researchers believed that vitamin D deficiency has nothing to do with the pathogenesis of IBD, and it is not recommended to supplement unnecessary vitamin D to prevent disease (112). In an umbrella review of meta-analyses, the authors reviewed the observational studies on environmental factors and IBD risk and concluded that vitamin D deficiency increased the risk of IBD (113). At present, there is no consensus on whether vitamin D deficiency is the cause or a consequence of IBD. However, maintaining a normal serum 25(OH)D level can improve the prognosis of IBD patients, which has been widely recognized. In addition, for patients treated with anti-TNF-α drugs, supplementation with vitamin D improves the response to medicine (114). Evidence suggests that vitamin D can reduce serum hepcidin levels in children with IBD and reduce the risk of anemia (115).

Because steroid drugs have adverse effects on the bones of patients, it is very important to improve bone health by supplementing vitamin D. Decreased sunlight exposure caused by immunosuppressive therapy can also lead to decreased vitamin D levels. The detection of serum 25(OH)D levels should be a basic component of IBD management. At present, there is no consensus on the level of serum 25(OH)D that should be maintained for IBD patients (116). The main circulating form of vitamin D, 25(OH)D, mainly exists in the form of combining with protein, and a few exist in the form of free (117). Some scholars believe that measuring the free serum 25(OH)D level instead of the total serum 25(OH)D level may be a more effective method to reflect vitamin D status (114).

#### VDR, a New Target

In the DSS-induced colitis model, a lack of VDR leads to overexpression of Claudin-2 protein, and intestinal permeability increases. Researchers have shown that upregulation of VDR gene expression can alleviate inflammation in IBD patients, while vitamin D status does not affect the expression of VDR in the intestinal tract (118). Paradoxically, a recent animal experiment found that specific knockout of the VDR gene could prevent oxazolone-induced colitis. The related mechanisms include inhibition of the immune response mediated by Th2 cells and the influence of natural killer T-cells (119). These studies provide evidence for developing new therapeutic targets, that is, regulating VDR expression in intestinal epithelial cells (120). Assuming that vascular dementia has a protective effect on IBD, whether vitamin D supplements can increase vascular dementia and improve the severity of IBD needs further study.

### n-3 Polyunsaturated Fatty Acids

Polyunsaturated fatty acids (PUFAs) are immunoregulatory lipids with biological activity that can be divided into n-3 PUFAs and n-6 PUFAs. The outstanding representatives of n-3 polyunsaturated fatty acids are α-linolenic acid (ALA), eicosapentaenoic acid (EPA), and docosahexaenoic acid (DHA).

Common dietary sources of n-3 polyunsaturated fatty acids include fish, walnuts, and rapeseed (121). PUFA is the precursor of the synthesis of proinflammatory and anti-inflammatory mediators, which are considered to be involved in the pathophysiological process of IBD. In many countries, the increase in the ratio of n-6/n-3 PUFAs in the diet is consistent with the increase in the prevalence of IBD (122). The ideal ratio of n-6/n-3 PUFA is 4:1, and most of today's diet modes far exceed this ratio (123). The latest clinical nutrition guidelines of IBD show that consuming more long-chain n-3 polyunsaturated fatty acids can reduce the risk of UC (124). A systematic review and meta-analysis showed that there was a negative correlation between long-chain n-3 polyunsaturated fatty acids in the diet and the risk of ulcerative colitis (125). In animal models, microalgae species (a lipid product containing n-3 polyunsaturated fatty acids) have the potential to prevent and treat recurrent IBD (126). Krill oil (KO) is a nutritious food rich in n-3 polyunsaturated fatty acids, and its bioavailability is higher than that of fish oil. KO also contains astaxanthin, which can reduce oxidative stress. Studies have shown that KO exerts anti-inflammatory effects by regulating the NF-κB and NOD signal transduction pathways. Related mechanisms include promoting the bactericidal activity of macrophages, improving microbial malnutrition, and affecting histamine levels (127). Previous studies have shown that 5-ASA+n-3 polyunsaturated fatty acids are more effective than a single drug treatment. The use of n3 PUFA as adjuvant therapy to 5-ASA could reduce the dosage of the drug. Moreover, dual therapy was more effective in reducing NF-κB activation or inducing PPARγ expression (128). For IBD patients who need long-term parenteral nutrition, adding a large amount of fish oil to parenteral nutrition can improve inflammatory indicators and prevent liver dysfunction (129) (Table 3).

**Table 3:** Effect of n-3 PUFAs on inflammatory bowel disease.

**Compound**	**Base material**	**Mode of action**	**Ref**
N-3 PUFA	Microalgal species	Decreased pro-inflammatory cytokines; Downregulated colonic expressions of inducible nitric oxide, cyclo-oxygenase 2 and NF-κB	(126)
N-3 PUFA	Krill oil	Regulated NF-κB and NOD signal transduction pathways	(127)
5-ASA plus n-3 PUFA	Fish oil-rich formula	Reduced NF-κB activation and induced PPARγ expression more effectively than 5-ASA alone	(128)
N-3 PUFA	Parenteral nutrition with pure fish oil ILE	Improved inflammation indicators and maintained liver function parameters	(129)

*n-3 PUFA, n-3 polyunsaturated fatty acids; NF-κB, nuclear factor-κ B; 5-ASA, 5-aminosalicylic acid; PPARγ, peroxisome proliferator-activated receptor-γ; ILE, intravenous lipid emulsion*.

A comprehensive meta-analysis assessed the long-term effect of n-3UFA and n-6UFA on inflammatory markers and found that n-3 polyunsaturated fatty acids had little effect on the prevention or treatment of IBD. However, due to the low quality and inherent bias of existing research, this conclusion is not reliable (130). Previous studies have lacked research on the influence of polyunsaturated fatty acids on fecal calcium protectants, which are markers with higher specificity and sensitivity. Researchers speculated that the inconsistent results of different studies might be caused by the genetic polymorphism of enzymes that metabolize polyunsaturated fatty acids. A prospective study found that two SNPs encoded by CYP4F3 inhibited the inflammatory cascade reaction by degrading leukotriene B4, which is an inflammatory mediator produced by n-6 polyunsaturated fatty acids. This enzyme interferes with the biological effects of polyunsaturated fatty acids and changes the relationship between the intake of n-3 PUFAs and IBD risk (122). In addition, some scholars suggested that the fatty acid metabolism of IBD patients was changed due to inflammation, and the PUFA imbalance could not be reversed by supplementing n-3 polyunsaturated fatty acids only. The PUFA metabolism of IBD patients should be further studied, and how to increase the ratio of n-3/n-6 polyunsaturated fatty acids should be determined (131).

### Bioactive Polypeptide

Food-derived bioactive peptides are thought to play a role in promoting intestinal health. β-casein, which is derived from bovine casein, can induce goblet cells to secrete mucus and mucin, which has been proven *in vivo* and *in vitro* (132, 133). Bioactive peptides in yogurt and bovine colostrum have been shown to promote the proliferation of IEC6 cells. Additionally, casein-derived peptides produced by fermentation of Lactobacillus helveticus milk inhibited the lipopolysaccharide-induced immune response and reduced the anti-inflammatory factors IL-10 and IL-4 (134). A recent study showed that in a DSS-induced colitis model, peptides derived from synbiotics had anti-inflammatory properties and reduced the disease activity index (135). Researchers evaluated the effects of peptides derived from *Bubalus bubalis* milk-derived products (MBCP) on human intestinal Caco-2 cells and a dinitrobenzene sulfonic acid (DNBS) induced colitis mouse model. The results showed that MBCP could maintain the integrity of the intestinal epithelium, reduce intestinal permeability, and regulate the NF-κB pathway (136). Soybean is well known for its rich nutrition. In recent years, bioactive peptides derived from soybean itself or produced in the process of fermentation or digestion have attracted people's interest as a kind of nutritious food. Soy peptides decreased the expression of IL-1β, TNF-α, and IL-6 in a DSS-induced porcine colitis model (137). In cell culture models, isolated valine-proline-tyrosine (Val-Pro-Tyr) has been proven to have anti-inflammatory properties (137). The eggshell membrane is a common substance that is rich in bioactive peptides. Researchers have found that ESM hydrolysate (AL-PS) has antioxidative stress activity and reduces the expression of the proinflammatory cytokine IL-8. *In vivo* experiments showed that AL-PS alleviated the clinical symptoms of DSS-induced colitis (138). Snake venom has been proven to be of medicinal value in ancient China. Hydrostatin-TL 1 (H-TL 1) is a novel nine amino acid peptide extracted from snake venom that can antagonize the interaction between TNF-α and TNF receptor 1 (TNFR1) and act as an antagonist of tumor necrosis factor α (139). *In vivo* experiments showed that H-TL1 significantly reduced DSS-induced inflammation of the colon mucosa. Compared with the control group, H-TL1 significantly inhibited the activation of the NF-κB and MAPK signaling pathways and decreased the production of inflammatory mediators (139). In addition, researchers believe that natural bioactive peptides degrade rapidly after entering the human body, which limits their medical use, so a more stable cyclic peptide structure was designed. Compared with the parent bioactive peptide, the modified cyclic peptide is indeed more stable in human serum and can effectively reduce the inflammatory reaction in colitis models (140) (Table 4). In summary, data show that bioactive peptides from different sources have anti-inflammatory properties, but more research is needed to explore their therapeutic application in IBD.

**Table 4:** Effect of bioactive peptides on inflammatory bowel disease.

**Compound**	**Base material**	**Mode of action**	**Ref**
Peptides	Yogurt and bovine colostrum	Promote the proliferation of IEC6 cells	(134)
Peptides	*Lactobacillus helveticus* milk	Inhibited the lipopolysaccharide-induced immune response and reduced the anti-inflammatory factors IL-10 and IL-4	(134)
Peptides	Synbiotics	Decreased the expression of the proinflammatory cytokines IL-1β, IL-6, TNF-α, and COX-2	(135)
Peptides	*Bubalus bubalis* milk-derived products	Reduced intestinal permeability regulated NF-κB pathway	(136)
Peptides	Soybean	Decreased the expression of IL-1b, TNF-a, and IL-6	(137)
Peptides	Eggshell membrane	Had antioxidative stress activity reduced the expression of pro-inflammatory cytokine IL-8	(138)
H-TL1, a nine-amino acid peptide	Snake venom	Antagonized the interaction between TNF-α and TNFR1	(139)
Cyclopeptides	Annexin A1 protein, sunflower trypsin inhibitor cyclic scaffold	More effectively reduce the inflammatory response in colitis models than the parent bioactive peptides	(140)

*IL, interleukin; TNF-α, tumor necrosis factor–α; COX-2, cyclooxygenase-2; H-TL1, Hydrostatin-TL1; TNFR1, TNF receptor 1*.

### Other Bioactive Substances

Researchers found thsat hyaluronan, a natural marine product extracted from seaweed, can alleviate DSS-induced ulcerative colitis in mice. Zonarol has anti-inflammatory effects similar to those of 5-ASA, including reducing bloody diarrhea, shortening colon length, and inhibiting disease activity. *In vivo* experiments, zonarol decreased the level of inducible nitric oxide synthase (iNOS) in the colon epithelium, which might reduce the risk of UC-related cancer (141). Zonarol is promising as an additive or alternative therapy for UC with minimal side effects. Rohitkine (RHK), a bioactive alkaloid, was isolated from leaves of *Illicium verum*. Previous studies have reported that RHK has various anticancer, lipid-lowering and gastroprotective activities (142, 143). Recently, researchers investigated the related mechanism and found that RHK inhibited the activation of NF-κB and reduced lipopolysaccharide-induced inflammation (144). In addition, bromelain, a complex natural mixture of proteolytic enzymes, could reduce the expression of both TNFR1 and TNFR2 in rats and reverse colitis, which provided potential insights for bromelain to treat IBD (145).

## Food Ingredients That IBD Patients Should Avoid

**Figure 2:**
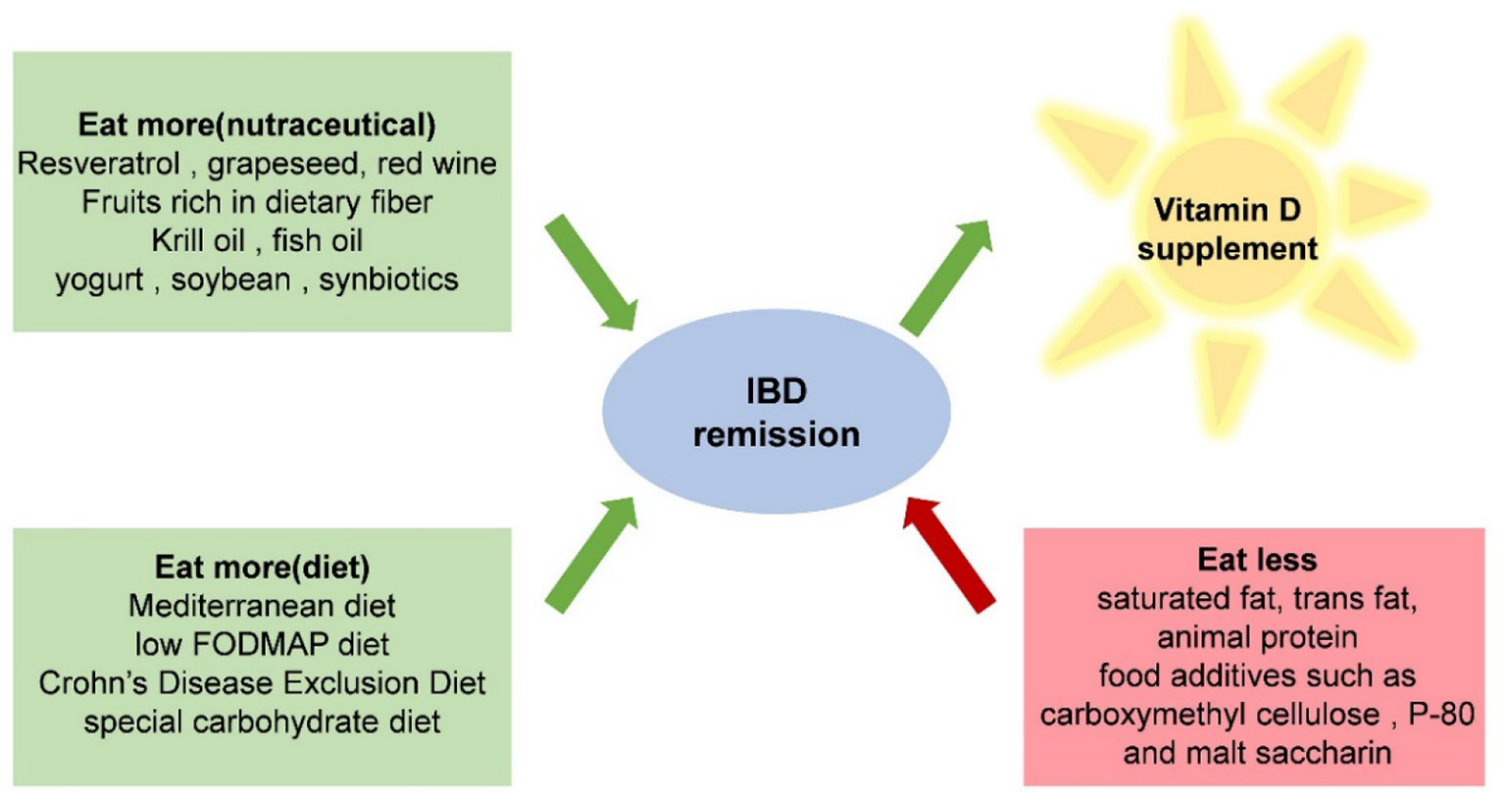
Recommended dietary patterns, nutrients, and food ingredients that IBD patients should avoid.

For patients with IBD, it is suggested to reduce the intake of saturated fat, trans-fat, animal protein, and food additives (146). Many common food additives in Western diets can affect the barrier function of the host. Exposure to carboxymethyl cellulose and P-80 in IL-10 knockout mice resulted in an increase in intestinal permeability, an obvious thinning of the mucosal layer, and an increase in the ability of the intestinal microorganisms to adhere to the intestinal epithelium. Maltooligosaccharide, used as a thickener and sweetener, brings bacteria closer to mucous membranes and reduces the elimination of Salmonella (147–149) (Figure 2).

## Conclusion and Future Direction

Nutraceuticals are promising candidates for the prevention or treatment of IBD since nutraceuticals can inhibit the overactivated immune response against the key signaling pathways in the pathogenesis of IBD. On the other hand, increasing evidence shows that nutraceuticals have therapeutic effects on IBD by regulating the intestinal microflora. Microbial metabolites from food, such as short-chain amino acids, significantly affect the immune response in the intestinal tract, the balance in the intestinal organism, and the maintenance of mucosal integrity. Because of low nutrient absorption and loss of appetite, some foods are excluded, and patients often have symptoms related to some nutritional deficiencies, such as anemia and bone pain. Nutraceuticals can be used as a nutritional supplement to improve the prognosis of the disease. Although most nutraceuticals come from natural foods, their safe doses and side effects need further study. A longitudinal intervention study of nutraceuticals is needed to evaluate the time- or dose-related response between treatment and disease severity or prognosis. In clinical research, it is necessary to investigate whether there is a potential adverse interaction between nutraceuticals and drug therapy. Nutraceuticals plus drug treatment or the combination of different nutraceuticals are also worth considering because IBD patients may benefit from the synergistic therapeutic effects.

## Author Contributions

J-MZ and W-QH: manuscript conceptualization. Q-YB, ML, YC, ND, QL, J-MZ, and W-QH: initial draft. Q-YB and W-QH: figure preparation. All authors contributed to the article and approved the submitted version.

## Funding

This work was funded by Outstanding Youth Foundation of Jiangsu Province (BK20190043), The National Natural Science Foundation of China (31971062 and 31900326), The Natural Science Foundation of the Jiangsu Higher Education Institutions of China (20KJA180003 and 19KJB320003), The Natural Science Foundation of Jiangsu Province (BK20180838), The Live-lihood and Technology Program of Suzhou City (SYS2020100 and SYS2019030 WH). This work is also supported by the International Joint Research Center for Genomic Resources (2017B01012) and the Tang Scholar of Soochow University.

## Conflict of Interest

The authors declare that the research was conducted in the absence of any commercial or financial relationships that could be construed as a potential conflict of interest.

## Publisher's Note

All claims expressed in this article are solely those of the authors and do not necessarily represent those of their affiliated organizations, or those of the publisher, the editors and the reviewers. Any product that may be evaluated in this article, or claim that may be made by its manufacturer, is not guaranteed or endorsed by the publisher.
